# Certain Topological Indices of Non-Commuting Graphs for Finite Non-Abelian Groups

**DOI:** 10.3390/molecules27186053

**Published:** 2022-09-16

**Authors:** Fawad Ali, Bilal Ahmad Rather, Muhammad Sarfraz, Asad Ullah, Nahid Fatima, Wali Khan Mashwani

**Affiliations:** 1 School of Mathematics and Statistics, Xi’an Jiaotong University, Xi’an 710049, China; 2 Institute of Numerical Sciences, Kohat University of Science & Technology, Kohat 26000, Pakistan; 3 Mathematical Sciences Department, College of Science, United Arab Emirate University, Al Ain 15551, United Arab Emirates; 4 Department of Mathematics, Sun Yat-sen University, Guangzhou 510275, China; 5 Department of Mathematical Sciences, University of Lakki Marwat, Lakki Marwat 28420, Pakistan; 6 Department of Mathematics and Sciences, Prince Sultan University, Riyadh 11586, Saudi Arabia

**Keywords:** non-commuting graphs, molecular structure, finite groups, topological index, Hosoya polynomial

## Abstract

A topological index is a number derived from a molecular structure (i.e., a graph) that represents the fundamental structural characteristics of a suggested molecule. Various topological indices, including the atom-bond connectivity index, the geometric–arithmetic index, and the Randić index, can be utilized to determine various characteristics, such as physicochemical activity, chemical activity, and thermodynamic properties. Meanwhile, the non-commuting graph $\Gamma_{G}$ of a finite group G is a graph where non-central elements of G are its vertex set, while two different elements are edge connected when they do not commute in G. In this article, we investigate several topological properties of non-commuting graphs of finite groups, such as the Harary index, the harmonic index, the Randić index, reciprocal Wiener index, atomic-bond connectivity index, and the geometric–arithmetic index. In addition, we analyze the Hosoya characteristics, such as the Hosoya polynomial and the reciprocal status Hosoya polynomial of the non-commuting graphs over finite subgroups of SL(2,C). We then calculate the Hosoya index for non-commuting graphs of binary dihedral groups.

## 1. Introduction

In a broad sense, molecular descriptors are a method for describing and quantifying a chemical composition using mathematics and cheminformatics techniques. It is necessary to understand that no molecular descriptor applies to all applications. Different descriptors can be used to study and describe the same molecule, depending on the question to be answered and the goals to be reached. There are several types of molecular descriptors, some of which use chemical graph theory [1]. These include chemical indices, topological indices, autocorrelation descriptors, geometrical descriptors, and certain molecular fingerprints. Most of them are useful for Computer-Assisted Structure Elucidation (CASE): to evaluate the topology and geometry between the data source and desired molecules; to easily determine identical features between a large number of chemical graphs, and to enable rapid scanning of chemical libraries based on essential molecular characteristics. Topological indices are two-dimensional molecular descriptors depending on the graph representation of the topology of the molecular structure. The molecular graph is the first topological index, representing a molecule in two dimensions. The first topological index is the molecular graph, which is a 2D graph that shows how a molecule appears. The molecular graph is a sparse, undirected, and weighted multigraph. When a chemical structure is shown as a graph, well-known tools from graph theory can be used to find important topological properties. Quantitative structure–property relationships (QSAR) are prediction models that use statistical methods to link the biological activity of chemicals with their molecular structure. In addition to lead optimization and drug discovery, QSARs are utilized in various fields, including regulatory decisions, toxicity prediction, and risk assessment [2].

Physicochemical properties (that is, the strain energy, stability, and boiling point) and topological indices (that is, the atom-bond connectivity (ABC) index, the Randić index, and the arithmetic–geometric (GA) index) are used in QSPR studies to determine the bioactivity of chemical compounds. Indeed, a topological index is produced by converting a chemical structure into a numeric number. It is a particular value which measures the symmetry of a molecule structure and determines its topology, while remaining unaffected by a function that retains the structure [3]. Several topological indices can be used to explore specific properties of molecularly structured chemical substances. In 1947, while investigating the boiling temperature of paraffin, Wiener constructed the first topological index, known as the path number [4]. Consequently, the Wiener index was invented, which originated the notion of a topological index. Several distance and degree-based topological indices have been introduced and discussed in the past few years, such as [5,6].

In order to determine the molecular orbital of unsaturated hydrocarbons, renowned chemists use Pólya’s [7] technique for computing polynomials. Hosoya [8] invented this notion in 1988 in order to compute the polynomials of several significant chemical graphs; this concept is known as the Hosoya polynomial. In 1996, the authors of [9] called the Hosoya polynomial the Wiener polynomial, although several researchers continue to refer to it as the Hosoya polynomial, which is often employed to identify distance-based graph characteristics. Cash established a connection among the Hosoya polynomial and the hyper Wiener index in [10]. Estrada et al. [11] concentrated on several unique applications of generalized Wiener index.

In this article, we will explore simple graphs with no loops or various edges. Assume that G is a finite non-abelian group. The non-commuting graph $\Gamma_{G}$ is a graph on G, where non-central elements of G are its vertex set such that two different elements are edge connected when they do not commute in G. Neumann [12] derived the concept of non-commuting graphs in 1975 by posing the challenge of determining the maximum clique of such a graph. Other scholars have also explored non-commuting graphs over finite groups, see [13,14] and the references therein. Further, the researchers have also examined commuting graphs in different context, such as groups of matrices [15,16], dihedral groups [17,18,19], and commutative rings with zero-divisors [20,21].

The authors of [22] demonstrated that the commuting graphs of the symmetric group Sym(n) and the alternating group Alt(n) on *n* letters, respectively, are either disconnected or have a diameter of no more than 5. In the same article, they conjectured that the diameter of a commuting graph has an absolute upper limit. This problem was eventually answered in [23], which showed an unlimited set of exceptional two-groups with increasing dimension commuting graphs. Numerous scholars have examined the connectivity and the metric dimensions of (non) commuting graphs over various finite groups; for instance, see [24] and their references.

A matching consists of a collection of edges with no shared vertices. A vertex that coincides with one of the matching edges is said to be matched. Otherwise, there exists an unmatched vertex. The Hosoya index or Z-index represents a graph’s most significant number of matchings. In 1971, Hosoya [25] conceived the Hosoya index and expanded it as a generic tool for physical chemistry [26]. It has been proven successful in various chemical problems, including measuring the heat of vaporization, entropy, and boiling point. The Hosoya index is a prime example of a topological index with significant applications in combinatorial chemistry. Numerous scholars explored a wide range of graph configurations when examining extremal problems associated with the Hosoya index.

Using the specified bounds, the authors of [27] analyzed the commuting involution graphs in special linear groups. The disc diameter of special linear groups in two and three dimensions are calculated. In addition, they provided examples of dimensionless commuting involution graphs. In [28], the authors investigated the Hosoya characteristics of non-commuting graphs of dihedral groups. In [5], the authors analyzed the Hosoya properties of power graphs of various finite groups. Several types of topological indices have been applied to commuting graphs related to finite groups, for instance, in [29,30], while the authors of [31] studied several topological indices of the non-commuting graphs over dihedral and generalized quaternion groups, respectively. Motivated by their work (as mentioned above), we devote ourselves to the non-commuting graphs of finite subgroups SL(2,C). It is very complicated to calculate the topological indices of $\Gamma_{G}$ for any finite group G. So, in this article, we focus our attention to examine several topological indices (as stated in Table 1), the (reciprocal status) Hosoya polynomial and the Hosoya index of a finite groups.

There are still significant gaps in the existing work about the identification of certain topological properties, the (reciprocal status) Hosoya polynomials as well as the Hosoya index of non-commuting graphs of finite subgroups of SL(2,C). The apparent explanation is that neither the construction of non-commuting graphs over finite groups nor the derivation of handy formulas of graph characteristics for comprehensive classes of groups. We make an attempt in this article to examine one of these problems

This article is structured as follows: Section 2 covers some findings and essential definitions that are useful to this article. Section 3 explores the construction of edge and vertex partitions. Various topological properties of non-commuting graphs over binary dihedral groups are demonstrated in Section 4. Section 5 discusses the Hosoya properties, that is, the Hosoya and its reciprocal status, and the Hosoya index of the non-commuting graph for finite subgroups of SL(2,C). Section 6 contains the conclusion and future work of the article.

## 2. Preliminaries

This section summarizes numerous basic graph-theoretic features and notable results that will be discussed in more detail later in this paper.

Assume that Γ is an undirected simple graph. The edge and vertex sets of Γ are denoted by E(Γ) and V(Γ), respectively. The order of Γ is the total number of vertices represented by |Γ|. The distance between vertices $u_{1}$ and $u_{2}$ in Γ, denoted by $\mathrm{dis}(u_{1},u_{2})$, is defined as the shortest path in both nodes $u_{1}$ and $u_{2}$. Two vertices $v_{1}$ and $v_{2}$ are connected if they share an edge, and it is represented by $v_{1}∼v_{2},$ otherwise $v_{1}≁v_{2}.$ N(x) represents the neighborhood of *x*, which consists of all vertices in Γ adjacent to *x*. The degree (valency) denoted by $d_{u_{1}}$ of $u_{1}$ is the set of vertices in Γ, that are edge connected to $u_{1}$, and the degree sum of a vertex *u* is $S_{u}=\sum_{v\in N(u)}d_{v}$. A $u_{1}-u_{2}$ path having $\mathrm{dis}(u_{1},u_{2})$ length is known as a $u_{1}-u_{2}$ geodesic. The greatest distance between $u_{1}$ and any other vertex in Γ is referred to as the eccentricity, and it is represented by $\mathrm{ec}(u_{1})$. Amongst every vertex in Γ, the diameter denoted by diam(Γ) has the highest eccentricity. Additionally, amongst every vertex of Γ, the radius rad(Γ) has the smallest eccentricity. Furthermore, a vertex $u_{1}$ is said to be a central vertex of Γ, if ec(Γ)=rad(Γ) and a vertex $u_{1}$ is called peripheral vertex, if ec(Γ)=diam(Γ). A subgraph induced by the central vertices and peripheral vertices of Γ are called centre and periphery, respectively. A graph Γ is known as self-centered if rad(Γ)=diam(Γ).

Suppose $\Gamma_{1}$ and $\Gamma_{2}$ are two connected graphs, then $\Gamma_{1}\vee \Gamma_{2}$ is the join of $\Gamma_{1}$ and $\Gamma_{2}$ whose edge and vertex sets are $E\left(\Gamma_{1}\right)\cup E\left(\Gamma_{2}\right)\cup \left\{y∼z:y\in V\left(\Gamma_{1}\right),\;z\in V\left(\Gamma_{2}\right)\right\}$ and $V\left(\Gamma_{1}\right)\cup V\left(\Gamma_{2}\right)$, respectively. A complete graph is one in which each individual vertex in the graph has an edge, and it is denoted by $K_{n}$. A graph that has its vertices partitioned into *k* different independent sets is said to be *k*-partite, and a complete *k*-partite graph contains an edge between any two vertices from different independent sets. Additional undefined expressions and symbols were obtained from [39].

**Definition** **1.** *Assume that G is a group. Then the centre of G is described as follows:*

$$Z\left(G\right)=\left\{g_{1}:g_{1}\in G\;\mathrm{and}\;g_{1}g_{2}=g_{2}g_{1},\;for\;all\;g_{2}\in G\right\}.$$

**Proposition** **1** ([13]). *For every non-ableian group G, we have $\mathrm{diam}(\Gamma_{G})=2$.*

As ec(u)≤2 for each $u\in \Gamma_{G}$, so we have the following proposition.

**Proposition** **2** ([30]). *A graph $\Gamma_{G}$ is self-centered, where G is a non-abelian group, and if for each $u\in \Gamma_{G}$, we have ec(u)=2. However, it is equal to the sum of the periphery and the center of $\Gamma_{G}$.*

The number of conjugacy classes in a group G is represented by the symbol κ(G), while $Z_{n}$ is used to denote the cyclic group of order n. The set of 2×2 matrices whose determinant is one forms the special linear group SL(2,C) of degree 2 over the complex field C. Moreover, the presentation of the binary dihedral group ${\mathrm{BD}}_{4n}$ of order 4n is defined as:

$${\mathrm{BD}}_{4n}=\langle y,z\;|\;y^{2n}=1,\;y^{n}=z^{2},\;zyz^{-1}=y^{-1}\rangle .$$

We now divide ${\mathrm{BD}}_{4n}$ as follows:

$$\Omega =\left\{e,y^{n}\right\},\;X_{1}=\langle y\rangle ,\;X_{2}=\overset{n-1}{\underset{i=0}{⋃}}X_{2}^{i},\;\mathrm{where}\;X_{2}^{i}=\left\{y^{i}z,\;y^{n+i}z\right\}\;\mathrm{and}\;X_{3}=X_{1}\setminus \Omega .$$

Therefore, there are n+3 conjugacy classes of ${\mathrm{BD}}_{4n}$. Furthermore,

$${\mathrm{BT}}_{24}=\langle r,s,t\;|\;r^{2}=s^{3}=t^{3}=rst\rangle ,$$

$${\mathrm{BO}}_{48}=\langle r,s,t\;|\;r^{2}=s^{3}=t^{4}=rst\rangle ,$$

$${\mathrm{BI}}_{120}=\langle r,s,t\;|\;r^{2}=s^{3}=t^{5}=rst\rangle ,$$

represents the binary tetrahedral group of order 24, the binary octahedral group of order 48, and the binary icosahedral group of order 120, respectively. All the mentioned above are finite non-abelian subgroups of SL(2,C).

Several characteristics of the mentioned groups will be investigated, but the non-commuting graph of ${\mathrm{BD}}_{4n}$ will be our prime motive. Hence, using GAP [40] calculations, we obtain Propositions 1 and 2, so we deduce the subsequent result, that is, the classification of the non-commuting graphs of finite subgroups of SL(2,C).

**Proposition** **3.** *The non-commuting graphs of finite subgroups of SL(2,C) have the following structure:*

$$\begin{array}{cc} \Gamma_{{\mathrm{BD}}_{4n}} & =K_{\underset{n-times}{\underset{︸}{2,2,\ldots ,2}},\left|X_{3}\right|};\\ \Gamma_{{\mathrm{BT}}_{24}} & =K_{2,2,2,\underset{4-times}{\underset{︸}{4,\ldots ,4}}};\\ \Gamma_{{\mathrm{BO}}_{48}} & =K_{6,6,6,\underset{6-times}{\underset{︸}{2,\ldots ,2}},\underset{4-times}{\underset{︸}{4,\ldots ,4}}};\\ \Gamma_{{\mathrm{BI}}_{120}} & =K_{\underset{15-times}{\underset{︸}{2,\ldots ,2}},\underset{10-times}{\underset{︸}{4,\ldots ,4}},\underset{6-times}{\underset{︸}{8,\ldots ,8}}}. \end{array}$$

According to the above classification, we obtain the following points of the non-commuting graph $\Gamma_{{\mathrm{BD}}_{4n}}$ of ${\mathrm{BD}}_{4n}$:

1. For $w_{1},w_{2}\in V\left(\Gamma_{{\mathrm{BD}}_{4n}}\right),w_{1}≁w_{2}$ when $w_{1},w_{2}\in X_{2}^{i}$ for each 0≤i≤n−1.
2. For $w_{1},w_{2}\in V\left(\Gamma_{{\mathrm{BD}}_{4n}}\right),w_{1}≁w_{2}$ when $w_{1},w_{2}\in X_{3}$.
3. For $w_{1},w_{2}\in V\left(\Gamma_{{\mathrm{BD}}_{4n}}\right),w_{1}≁w_{2}$ when $w_{1}\in X_{2}$ and $w_{2}\in X_{3}$.
4. For $w_{1},w_{2}\in V\left(\Gamma_{{\mathrm{BD}}_{4n}}\right),w_{1}≁w_{2}$ when $w_{1}\in X_{2}^{i}$ and $w_{2}\in X_{2}^{j}$ with i≠j and 0≤j,i≤n−1.
5. It can be observed in $\Gamma_{{\mathrm{BD}}_{4n}}$ that $\mathrm{ec}(w_{2})=2$ for every $w\in X_{2}\cup X_{3}$. As a result, $\Gamma_{{\mathrm{BD}}_{4n}}$ is a self-centered graph, that is equivalent to $K_{\underset{n-times}{\underset{︸}{2,2,\ldots ,2}},\left|X_{3}\right|}$ having *n*-partite sets $X_{2}^{i},$ where 0≤i≤n−1, and one partite set $X_{3}$.

The following relevant properties for the non-commuting graph $\Gamma_{G}$ was suggested in [13,30].

## 3. Edge and Vertex Partitions

To begin, we develop a number of interesting components that help in the evaluation of certain topological indices. The following parameters are defined for each *u* of Γ:

1. In Γ, the total distance number of *w* is $D\left(w|\Gamma \right)=\sum_{x\in V(\Gamma )}\mathrm{dis}\left(x,w\right))$.
2. In Γ, *w*’s total reciprocal distance is $D_{r}\left(w|\Gamma \right)=\sum_{x\in V(\Gamma )}\frac{1}{\mathrm{dis}(x,w)}$.
3. The sum of *w*’s distances in Γ is $D_{s}\left(w|\Gamma \right)=\sum_{x\in V(\Gamma )\setminus \{w\}}\frac{1}{\left(\mathrm{diam}(\Gamma )+1-\mathrm{dis}(w,x)\right)}$.

Table 1 contains the distance-based topological indices became:

(1) $$\mathrm{RCW}\left(\Gamma \right)=\frac{1}{2}\sum_{w\in V(\Gamma )}D_{s}\left(w|\Gamma \right)+\frac{\left|\Gamma \right|}{\mathrm{diam}(\Gamma )+1},$$

(2) $$\mathrm{MTI}\left(\Gamma \right)=\sum_{w\in V(\Gamma )}{\left(d\left(w\right)\right)}^{2}+\sum_{w\in V(\Gamma )}d\left(w\right)D\left(w|\Gamma \right),$$

(3) $$H\left(\Gamma \right)=\frac{1}{2}\sum_{w\in V(\Gamma )}D_{r}\left(w|\Gamma \right).$$

## 4. Topological Properties

Several topological characteristics of non-commuting graphs associated with binary dihedral groups are discussed in this section.

**Theorem** **1.** *Suppose $\Gamma_{{\mathrm{BD}}_{4n}}$ is a non-commuting graph of ${\mathrm{BD}}_{4n}$. Then:*

$$H\left(\Gamma_{{\mathrm{BD}}_{4n}}\right)=\frac{1}{2}\left(14n^{2}-16n+3\right).$$

**Proof.**  We have determined the Harary index by substituting the vertex partition, as mentioned in Table 2 and in Equation (3).

$$H\left(\Gamma_{{\mathrm{BD}}_{4n}}\right)=\frac{n(8n-7)}{2}+\frac{3(n-1)(2n-1)}{2}.$$

One may derive the required Harary index using a series of algebraic calculations. □

**Table 2:** Vertex partition of $\Gamma_{{\mathrm{BD}}_{4n}}$ for every $u\in V(\Gamma_{{\mathrm{BD}}_{4n}})$.

$d_{u}$	ec(u)	$D(u|\Gamma_{{\mathrm{BD}}_{4n}})$	$D_{s}\left(u|\Gamma_{{\mathrm{BD}}_{4n}}\right)$	$D_{r}\left(u|\Gamma_{{\mathrm{BD}}_{4n}}\right)$	Number of Vertices
4n−4	2	4n−2	2n−1	$\frac{1}{2}\left(8n-7\right)$	2n
2n	2	6n−6	3n−3	$\frac{3}{2}\left(2n-1\right)$	2(n−1)

**Theorem** **2.** *Assume that $\Gamma_{{\mathrm{BD}}_{4n}}$ is a non-commuting graph of ${\mathrm{BD}}_{4n}$. Then:*

$$H_{r}\left(\Gamma_{{\mathrm{BD}}_{4n}}\right)=\frac{n(11n-10)}{6n-4}.$$

**Proof.**  By applying the edge partition presented in Table 3 and the harmonic index in Table 1, we obtain:

$$H_{r}\left(\Gamma_{{\mathrm{BD}}_{4n}}\right)=\frac{4n(n-1)}{8(n-1)}+\frac{4n(n-1)}{3n-2}.$$

Certain computations result in the appropriate formula for the harmomic index. □

**Table 3:** Edge partition of $\Gamma_{{\mathrm{BD}}_{4n}}$ for any $u∼w\in E(\Gamma_{{\mathrm{BD}}_{4n}})$.

$\left(d_{v},d_{w}\right)$ Type Edges	$\left(S_{v},S_{w}\right)$ Type Edges	Edges Count
((4n−4),(4n−4))	((4n−4)(3n−2),(4n−4)(3n−2))	2n(n−1)
(2n,(4n−4))	(8n(n−1),(4n−4)(3n−2))	4n(n−1)

Note that (*d_v_*, *d_w_*) represents the kind of *v* ∼ *w* edge defined by the degrees of the end vertices, while (*S_v_*, *S_w_*) represents the kind of *v* ∼ *w* edge defined by the degrees sum of the end vertices.

**Theorem** **3.** *Suppose $\Gamma_{{\mathrm{BD}}_{4n}}$ is the non-commuting graph of ${\mathrm{BD}}_{4n}$ Then:*

$$R_{\alpha }\left(\Gamma_{{\mathrm{BD}}_{4n}}\right)=\left\{\begin{array}{cc} 32n{\left(n-1\right)}^{2}\left(2n-1\right), & \;for\;\alpha =1,\\ \frac{5n-4}{8(n-1)}, & \;for\;\alpha =-1,\\ 8n{\left(n-1\right)}^{2}+4n\left(n-1\right)\sqrt{8n(n-1)}, & \;for\;\alpha =\frac{1}{2},\\ \frac{4n\left(n-1\right)+2n\sqrt{8n(n-1)}}{\sqrt{8n(n-1)}}, & \;for\;\alpha =-\frac{1}{2}. \end{array}\right.$$

**Proof.**  Compute the edge partition, as shown in Table 3, using the generic Randić index $R_{\alpha }$ formula for $\alpha =1,-1,\frac{1}{2},-\frac{1}{2},$ we have:

$$\begin{array}{cc} R_{1}\left(\Gamma_{{\mathrm{BD}}_{4n}}\right) & =\frac{64n{\left(n-1\right)}^{2}\left(n-1\right)}{2}+32n^{2}{\left(n-1\right)}^{2},\\ \; & =32n{\left(n-1\right)}^{2}\left(2n-1\right).\\ R_{-1}\left(\Gamma_{{\mathrm{BD}}_{4n}}\right) & =\frac{n(n-1)}{2n^{3}-2n}+\frac{n(n-1)}{8{\left(n-1\right)}^{2}}.\\ R_{\frac{1}{2}}\left(\Gamma_{{\mathrm{BD}}_{4n}}\right) & =8n{\left(n-1\right)}^{2}+4n^{2}-4n\sqrt{8n(n-1)}.\\ R_{-(\frac{1}{2})}\left(\Gamma_{{\mathrm{BD}}_{4n}}\right) & =\frac{n(n-1)}{2(n-1)}+\frac{4n(n-1)}{\sqrt{8n(n-1)}}. \end{array}$$

We obtain the desired results by applying certain simplifications. □

**Theorem** **4.** *Suppose $\Gamma_{{\mathrm{BD}}_{4n}}$ is a non-commuting graph of ${\mathrm{BD}}_{4n}$. Then*

$$RCW\left(\Gamma_{{\mathrm{BD}}_{4n}}\right)=\frac{1}{3}\left(15n^{2}-17n+7\right).$$

**Proof.**  As $\Gamma_{{\mathrm{BD}}_{4n}}$ has a diameter of 2, so by computing the RCW index, we may use Equation (1) and the vertex partition as shown in Table 2.

$$RCW\left(\Gamma_{{\mathrm{BD}}_{4n}}\right)=3{\left(n-1\right)}^{2}+n\left(2n-1\right)+\frac{2(2n-1)}{3}.$$

We get the desire result by applying certain simplifications. □

**Theorem** **5.** *Assume that $\Gamma_{{\mathrm{BD}}_{4n}}$ is a non-commuting graph of ${\mathrm{BD}}_{4n}$. Then:*

$$\begin{array}{cc} \mathrm{ABC}\left(\Gamma_{{\mathrm{BD}}_{4n}}\right) & =\sqrt{12n{\left(n-1\right)}^{2}}+\frac{n\sqrt{8n-10}}{2}.\\ {\mathrm{ABC}}_{4}\left(\Gamma_{{\mathrm{BD}}_{4n}}\right) & =\sqrt{\frac{20n^{3}-28n^{2}+6n}{3n-2}}+\sqrt{\frac{2n^{2}\left(2n-1\right)\left(6n-7\right)}{3n-2}}. \end{array}$$

**Proof.**  By incorporating the edge partition presented in Table 3 into the ABC as well as ${\mathrm{ABC}}_{4}$ indices calculations, we obtain:

$$\begin{array}{cc} \mathrm{ABC}\left(\Gamma_{{\mathrm{BD}}_{4n}}\right) & =\left(4n^{2}-4n\right)\sqrt{\frac{6n-6}{8n(n-1)}}+\frac{4n\left(n-1\right)\sqrt{8n-10}}{8(n-1)},\;\;\mathrm{and}\;\\ {\mathrm{ABC}}_{4}\left(\Gamma_{{\mathrm{BD}}_{4n}}\right) & =\frac{\left(4n^{2}-4n\right)\sqrt{8(3n-2)(n-1)-2}}{8(n-1)(3n-2)}\\ \; & +\left(4n^{2}-4n\right)\sqrt{\frac{4(n-1)(3n-2)+8n(n-1)-2}{16n{\left(n-1\right)}^{2}\left(6n-4\right)}}. \end{array}$$

One may have the appropriate formulae for both indices by performing a simple simplification. □

**Theorem** **6.** *Assume that $\Gamma_{{\mathrm{BD}}_{4n}}$ is the non-commuting graph of ${\mathrm{BD}}_{4n}$. Then:*

$$\begin{array}{cc} \mathrm{GA}\left(\Gamma_{{\mathrm{BD}}_{4n}}\right) & =\frac{8n\left(3n-2\right)\left(n-1\right)+\sqrt[{3}]{8n(n-1)}}{2(3n-2)},\\ {\mathrm{GA}}_{5}\left(\Gamma_{{\mathrm{BD}}_{4n}}\right) & =\frac{n\left(n-1\right)\left(10n-4+\sqrt{64n(6n-4)}\right)}{5n-2}. \end{array}$$

**Proof.**  We have obtained the geometric arithmetic GA index and its 5th version by utilizing the formulae and the edge partition in Table 3.

$$\begin{array}{cc} \mathrm{GA}\left(\Gamma_{{\mathrm{BD}}_{4n}}\right) & =\frac{n^{2}-2n}{2}+\frac{2n\left(n-2\right)\sqrt{n(2n-4)}}{3n-4},\\ {\mathrm{GA}}_{5}\left(\Gamma_{{\mathrm{BD}}_{4n}}\right) & =2n^{2}-2n+\frac{4n\left(n-1\right)\sqrt{16n{\left(n-1\right)}^{2}\left(6n-4\right)}}{4n(n-1)+2(n-1)(6n-4)}. \end{array}$$

After several computations, the needed values of the GA index and its 5th version may be achieved. □

## 5. Hosoya Properties

The next section defines the Hosoya properties that are being considered and calculate them for finite subgroups of SL(2,C). We begin by computing the Hosoya polynomial, then determine its reciprocal status, and at last, we explore the Hosoya index.

### 5.1. Hosoya Polynomial

The first two results in this subsection give the coefficients required to build the Hosoya polynomial for the non-commuting graph on ${\mathrm{BD}}_{4n}$.

**Proposition** **4.** *Suppose $\Gamma_{{\mathrm{BD}}_{4n}}$ is the non-commuting graph that corresponds to ${\mathrm{BD}}_{4n}$. Then:*

$$\mathrm{dis}\left(\Gamma_{{\mathrm{BD}}_{4n}},u\right)=\left\{\begin{array}{cc} 2(2n-1), & \;whenever\;u=0;\\ 6n(n-1), & \;whenever\;u=1;\\ 2n^{2}-4n+3, & \;whenever\;u=2. \end{array}\right.$$

**Proof.**  Since we know that, $\mathrm{diam}\left(\Gamma_{{\mathrm{BD}}_{4n}}\right)=2$, then we want to determine $\mathrm{dis}\left(\Gamma_{{\mathrm{BD}}_{4n}},0\right),$$\mathrm{dis}\left(\Gamma_{{\mathrm{BD}}_{4n}},1\right)$ and $\mathrm{dis}\left(\Gamma_{{\mathrm{BD}}_{4n}},2\right).$ Suppose $V_{p}$ is the collection of every the pairs (distinct and same) of vertices of $\Gamma_{{\mathrm{BD}}_{4n}}$, then:

Vp=ΓBD4n2+ΓBD4n=2n(4n−3)+1.

Let

$$S\left(\Gamma_{{\mathrm{BD}}_{4n}},u\right)=\left\{\left(v_{1},v_{2}\right);v_{1},v_{2}\in V\left(\Gamma_{{\mathrm{BD}}_{4n}}\right)|\mathrm{dis}\left(v_{2},v_{2}\right)=u\right\}$$

and $\mathrm{dis}\left(\Gamma_{{\mathrm{BD}}_{4n}},u\right)=$$|S\left(\Gamma_{{\mathrm{BD}}_{4n}},u\right)|$. Then:

(4) $$V_{p}=S\left(\Gamma_{{\mathrm{BD}}_{4n}},0\right)\cup S\left(\Gamma_{{\mathrm{BD}}_{4n}},1\right)\cup S\left(\Gamma_{{\mathrm{BD}}_{4n}},2\right).$$

As, $\mathrm{dis}(v_{1},v_{1})=0,$ for every $v_{1}\in V\left(\Gamma_{{\mathrm{BD}}_{4n}}\right)$, then $S\left(\Gamma_{{\mathrm{BD}}_{4n}},0\right)=V\left(\Gamma_{{\mathrm{BD}}_{4n}}\right)$. Hence $\mathrm{dis}\left(\Gamma_{{\mathrm{BD}}_{4n}},0\right)=2\left(2n-1\right).$ Using Proposition 3, $\Gamma_{{\mathrm{BD}}_{4n}}$ has the representation $K_{\underset{n-times}{\underset{︸}{2,2,\ldots ,2}},\left|X_{3}\right|}$ with

$$V\left(K_{\underset{n-times}{\underset{︸}{2,2,\ldots ,2}},\left|X_{3}\right|}\right)=X_{2}\cup X_{3}.$$

Therefore:

$$S\left(\Gamma_{{\mathrm{BD}}_{4n}},1\right)=\left\{\left(v_{1},v_{2}\right);v_{1}\in X_{2},v_{2}\in X_{3}\right\}\cup \left\{\left(v_{1},v_{2}\right);v_{1},v_{2}\in X_{2}\right\}.$$

Accordingly

disΓBD4n,1=2n2−n+(n−1)4n=6n(n−1).

Using Equation (4), we obtain $\left|V_{p}\right|=\mathrm{dis}\left(\Gamma_{{\mathrm{BD}}_{4n}},0\right)+\mathrm{dis}\left(\Gamma_{{\mathrm{BD}}_{4n}},1\right)+\mathrm{dis}\left(\Gamma_{{\mathrm{BD}}_{4n}},2\right)$. Thus

$$\begin{array}{cc} \mathrm{dis}\left(\Gamma_{{\mathrm{BD}}_{4n}},2\right) & =\left|V_{p}\right|-\left(\mathrm{dis}\left(\Gamma_{{\mathrm{BD}}_{4n}},1\right)+\mathrm{dis}\left(\Gamma_{{\mathrm{BD}}_{4n}},0\right)\right)\\ & =2n\left(4n-3\right)+1-\left(6n(n-1)+2(2n-1)\right)\\ & =2n^{2}-4n+3. \end{array}$$

Combining them we get the required. □

The following result determines the Hosoya polynomials of $\Gamma_{{\mathrm{BD}}_{4n}}$.

**Theorem** **7.** *For any n≥2, the Hosoya polynomial of $\Gamma_{{\mathrm{BD}}_{4n}}$ is given as:*

$$H\left(\Gamma_{{\mathrm{BD}}_{4n}},x\right)=\left(2n^{2}-4n+3\right)x^{2}+\left(6n(n-1)\right)x+4n-2.$$

**Proof.**  Using the values of $\mathrm{dis}\left(\Gamma_{{\mathrm{BD}}_{4n}},u\right)$ from Propositions 4, we obtain the following formula presented in Table 1 for the Hosoya polynomial:

$$\begin{array}{cc} H\left(\Gamma_{{\mathrm{BD}}_{4n}},x\right) & =\mathrm{dis}\left(\Gamma_{{\mathrm{BD}}_{4n}},2\right)x^{2}+\mathrm{dis}\left(\Gamma_{{\mathrm{BD}}_{4n}},1\right)x^{1}+\mathrm{dis}\left(\Gamma_{{\mathrm{BD}}_{4n}},0\right)x^{0}\\ & =\left(2n^{2}-4n+3\right)x^{2}+\left(6n(n-1)\right)x+4n-2. \end{array}$$

□

**Theorem** **8.** *Assume that $\Gamma_{G}$ is the non-commuting graph of G. Then:*

$$\begin{array}{cc} \;If\;G & ={\mathrm{BT}}_{24},\;\mathrm{then}\;H\left(\Gamma_{G},x\right)=27x^{2}+204x+22.\\ \;If\;G & ={\mathrm{BO}}_{48},\;\mathrm{then}\;H\left(\Gamma_{G},x\right)=75x^{2}+960x+46.\\ \;If\;G & ={\mathrm{BI}}_{120},\;\mathrm{then}\;H\left(\Gamma_{G},x\right)=243x^{2}+6660x+118. \end{array}$$

**Proof.**  Using Proposition 3, GAP [40], and applying the same calculations as in Theorem 7, we can obtain the desired result. □

### 5.2. Reciprocal Status Hosoya Polynomial

To begin, we determine the reciprocal status of every vertex of the non-commuting graphs of finite subgroups of SL(2,C). Then discuss its reciprocal status Hosoya polynomial.

**Proposition** **5.** *If $w\in V(\Gamma_{{\mathrm{BD}}_{4n}})$, then*

$$rs\left(w\right)=\left\{\begin{array}{cc} \frac{8n-7}{2}, & \;when\;w\in X_{2},\\ \frac{3(2n-1)}{2}, & \;when\;w\in X_{3}. \end{array}\right.$$

**Proof.**  By Proposition 3, $\Gamma_{{\mathrm{BD}}_{4n}}=K_{\underset{n-times}{\underset{︸}{2,2,\ldots ,2}},\left|X_{3}\right|}$ with the vertex set $X_{2}\cup X_{3}$. Accordingly, we have:

Whenever $w\in X_{2}$, and as $X_{2}={⋃}_{i=0}^{n-1}X_{2}^{i}$, so for any 0≤i≤n−1dis(w,v)=1 when $v\in V\left(\Gamma_{{\mathrm{BD}}_{4n}}\right)-X_{2}^{i}$ and dis(w,v)=2 when $v\in X_{2}^{i}$. Consequently, by defining reciprocal status, we obtain

$$rs\left(v\right)=\left(\frac{1}{1}\right)\left\{2\left(n-1\right)+2n-2\right\}+\left(\frac{1}{2}\right)1=\frac{8n-7}{2}.$$

Whenever $w\in X_{3}\;:\;\mathrm{dis}(w,v)=1$, when $v\in X_{2}$ and dis(w,v)=2, when $v\in X_{3}\setminus \left\{w\right\}$. Thus, using the reciprocal status formula, we obtain

$$rs\left(v\right)=2n\left(\frac{1}{1}\right)+\left(\frac{1}{2}\right)\left(2n-3\right)=\frac{6n-3}{2}.$$

Combining them we obtain the required result. □

**Theorem** **9.** *For any n≥4, the reciprocal status Hosoya polynomial of $\Gamma_{{\mathrm{BD}}_{4n}}$ is given by:*

$$H_{rs}\left(\Gamma_{{\mathrm{BD}}_{4n}}\right)=2n\left(n-1\right)x^{8n-7}+4n\left(n-1\right)x^{7n-5}.$$

**Proof.**  According to Proposition 5, $\Gamma_{{\mathrm{BD}}_{4n}}$ has two kinds of edges (α∼β and α∼β) based on the reciprocal status of end vertices, whenever $\alpha =\frac{8n-7}{2}$ while $\beta =\frac{3(2n-1)}{2}$. The reciprocal Hosoya polynomial’s formula is provided in Table 1, and we can use the edge partition from Table 4 to obtain:

$$\begin{array}{cc} H_{rs}\left(\Gamma_{{\mathrm{BD}}_{4n}}\right) & =\sum_{E_{\alpha ∼\alpha }}x^{\alpha +\alpha }+\sum_{E_{\alpha ∼\beta }}x^{\alpha +\beta }\\ & =2n\left(n-1\right)x^{\left(\frac{8n-7}{2}\right)+\left(\frac{8n-7}{2}\right)}+4n\left(n-1\right)x^{\left(\frac{8n-7}{2}\right)+\frac{3}{2}\left(2n-1\right)}\\ & =2n\left(n-1\right)x^{8n-7}+4n\left(n-1\right)x^{7n-5}. \end{array}$$

This conclusively establishes the proof. □

**Table 4:** Edge partition of $\Gamma_{{\mathrm{BD}}_{4n}}$ for any $x∼y\in E(\Gamma_{{\mathrm{BD}}_{4n}})$.

Kind of Edge	Partition of the Edge Set	Counting Edges
v∼v	$E_{v∼v}=\left\{ab\in E\left(\Gamma_{{\mathrm{BD}}_{4n}}\right):\;rs\left(a\right)=v,rs\left(b\right)=v\right\}$	$|E_{v∼v}|=2n\left(n-1\right)$
v∼w	$E_{v∼w}=\left\{ab\in E\left(\Gamma_{{\mathrm{BD}}_{4n}}\right):\;rs\left(a\right)=v,rs\left(b\right)=w\right\}$	$|E_{v∼w}|=4n\left(n-1\right)$

**Theorem** **10.** *Assume that $\Gamma_{G}$ is the non-commuting graph of G. Then:*

$$\begin{array}{cc} \;If\;G & ={\mathrm{BT}}_{24},\;\mathrm{then}\;H_{rs}\left(\Gamma_{G},x\right)=12x^{41}+96x^{40}+48x^{39}.\\ \;If\;G & ={\mathrm{BO}}_{48},\;\mathrm{then}\;H_{rs}\left(\Gamma_{G},x\right)=48x^{87}+288x^{86}+72x^{85}+192x^{66}+216x^{65}+60x^{45}.\\ \;If\;G & ={\mathrm{BI}}_{120},\;\mathrm{then}\;H_{rs}\left(\Gamma_{G},x\right)=420x^{233}+1200x^{232}+144x^{231}+1440x^{230}+1920x^{229}+740x^{227}. \end{array}$$

**Proof.**  Using Proposition 5, GAP [40], and applying the same calculations as in Theorem 9, we can obtain the desired result. □

### 5.3. Hosoya Index

The Hosoya index of the non-commuting graph on ${\mathrm{BD}}_{4n}$ is investigated in this subsection. To begin, take a note of the total of non-empty matchings presented in Table 5 for $K_{m}$, whereas $\delta_{\tau }$ represents the total possible matchings of order τ, where 1≤τ≤n.

For any n≥2, the subsequent result calculates the Hosoya index of $\Gamma_{{\mathrm{BD}}_{4n}}$ on ${\mathrm{BD}}_{4n}$.

**Theorem** **11.** *The Hosoya index of $\Gamma_{{\mathrm{BD}}_{4n}}$ is given as:*

$$1+\sum_{\tau =1}^{2(n-1)}\delta_{\tau }^{1}+\sum_{\tau =1}^{n}\delta_{\tau }^{2}+\sum_{\tau =2}^{2n-1}\delta_{\tau }^{3},$$

*where*

δ11=4n(n−1)andfor2≤τ≤2n−2,δτ1=2(n−1)!(2n−2−τ)!2n2,δτ2=1τ∏i=0τ−12(n−i)2−(n−2i),δτ3=∑i=1τ−1δi1τ−i∏k=1τ−i(2n−(i+2(k−1))2)−φki,whereφki=n−124k+i−2.

**Proof.**  As a result of Proposition 3, it is clear that, $\Gamma_{{\mathrm{BD}}_{4n}}=N_{2n-2}+\sum_{i=1}^{n}N_{2}^{i}$. As a result, $\Gamma_{{\mathrm{BD}}_{4n}}$ has the succeeding two kinds of edges.

**Type-1:**  $uv\in E(\Gamma_{{\mathrm{BD}}_{4n}})$ for $u\in X_{3}$ and $v\in X_{2}$;
**Type-2:**  $uv\in E(\Gamma_{{\mathrm{BD}}_{4n}})$ for $u\in X_{3}^{i}$ and $v\in X_{2}^{\ell }$ for i≠ℓ.

As a consequence, there are 3 distinct forms of matchings among the $\Gamma_{{\mathrm{BD}}_{4n}}$ edges.

$M_{1}$:  Type-1 edge matchings;
$M_{2}$:  Type-2 edges matchings;
$M_{3}$:  Type-1 and Type-2 edge matchings

$M_{1}$:  If $\delta_{\tau }^{1}$ determines the number of order τ matchings, then the total possible order 1 matchings equals the number of Type-1 edges, which is 4n(n−1), i.e., $\delta_{1}^{1}=4n\left(n-1\right)$. Furthermore, we have
  $$\begin{array}{cc} \delta_{2}^{1} & =2n\left[1\times 1(2n-1)\times (2n-3)\right]+2n\left[1\times (4n-2)\times (n-2)\right]\\ \; & +2n\left[1\times 1(2n-5)(2n-1)\right]+\cdots \\ \; & +2n\left[1\times 1(2n-1)(2n-(\ell +2))\right],\;\mathrm{where}\;1\leq \ell \leq 2n-3\\ \; & =\left(4n^{2}-2n\right)\sum_{\ell =1}^{2n-3}\left(2n-\left(\ell +2\right)\right)\\ \delta_{3}^{1} & =2n\left[1\times 1\left(2n-1\right)\times 1\left(2(n-1)\right)\times \left(2(n-2)\right)\right]\\ \; & +2n\left[1\times 1\left(2n-1\right)\times 1\left(2(n-1)\right)\times \left(2n-5\right)\right]\\ \; & +2n\left[1\times 1\left(2n-1\right)\times 1\left(2(n-1)\right)\times \left(2(n-3)\right)\right]\\ \; & +\cdots +2n\left[1\times 1\left(2n-1\right)\times 1\left(2(n-1)\right)\times \left(2n-\left(\ell +3\right)\right)\right], \end{array}$$
  where 1≤ℓ≤2n−4
  $$=4n\left(2n-1\right)\left(n-1\right)\sum_{\ell =1}^{2n-4}\left(2n-\left(\ell +3\right)\right).\;\;\;\;\;\;$$
  In general, for any 2≤τ≤2(n−1),
  $$\delta_{\tau }^{1}=\prod_{i=0}^{\tau -1}\left(2n-i\right)\sum_{\ell =1}^{2n-(\tau +1)}\left(2n-\left(\ell +\tau \right)\right).$$
  Note that
  $$\begin{array}{cc} \prod_{i=0}^{\tau -1}\left(2n-i\right) & =(2n)(2n-1)\cdots (2n-(\tau -1))\\ \; & =\frac{(2n)!}{(2n-\tau )!} \end{array}$$
  and
  $$\begin{array}{cc} \sum_{\ell =1}^{2n-(\tau +1)}\left(2n-\left(\ell +\tau \right)\right) & =(2n-(\tau +1))+\cdots +(2n-(2n-2))+(2n-(2n-1))\\ & =\frac{\left(2n-\tau )(2n-\tau -1\right)}{2}. \end{array}$$
  Thus, for 2≤τ≤2(n−1),
  δτ1=(2n−τ)(2n−τ−1)22n!(2n−τ)!=2n2(2n−2)!(2n−τ−2)!.
$M_{2}$:  If $\delta_{\tau }^{2}$ signifies the total possible matchings of cardinality τ, then the total possible matchings of cardinality 1 equals the total Type-2 edges. It is worth noting that the Type-2 edges correspond to the edges of $\Gamma_{{\mathrm{BD}}_{4n}}$’s subgraph $K_{\underset{n-times}{\underset{︸}{2,2,\ldots ,2}}}$, which is isomorphic to $K_{2n}-ne$, where $K_{2n}-\tau e$ represents a graph formed by removing τ edges from $K_{2n}$. Therefore, the size of $K_{\underset{n-times}{\underset{︸}{2,2,\ldots ,2}}}$ is equal to the size of $K_{2n}-ne$, which is 2n2−n. Thus, based on the number of matches recorded for $K_{2n}$ in Table 5, we may calculate the possible matches for $\delta_{\tau }^{2}$, for 1≤τ≤n, given as:
  δ12=2n2−n,δ22=2n2−n2n−22−(n−2),δ32=2n2−n2n−22−(n−2)2n−42−(n−4),δ42=2n2−n2n−22−(n−2)2n−42−(n−4)2n−62−(n−6),⋮δτ2=1τ∏i=0τ−12n−2i2−(n−2i).

$M_{3}:\;$

If $\delta_{\tau }^{3}$ indicates the number of order τ matchings, then $\delta_{1}^{3}=0$. Following that, for 2≤τ≤2n−1, the possible matches may be determined as follows employing the product rule:
  δ23=δ112n−12−(n−1),δ33=δ11×122n−12−(n−1)2n−32−(n−3)+δ212n−22−(n−1),δ43=δ11×132n−12−(n−1)2n−32−(n−3)2n−52−(n−5)+δ21×122n−22−(n−2)2n−42−(n−4)+δ312n−32−(n−2),⋮δτ3=∑i=1τ−1δi1(τ−i)∏k=1τ−i(2n−(i+2(k−1))2)−φki,
  where $\varphi_{k}^{i}=n-\frac{1}{2}\left(4k+i-2\right).$

Thus, $\Gamma_{{\mathrm{BD}}_{4n}}$ has the following Hosoya index:

$$1+\sum_{\tau =1}^{2n-2}\delta_{\tau }^{1}+\sum_{\tau =1}^{n}\delta_{\tau }^{2}+\sum_{\tau =2}^{2n-1}\delta_{\tau }^{3}.$$

□

## 6. Conclusions and Future Work

This article aimed to examine the structural properties of non-commuting graphs of finite non-abelian groups. Such groups have a well-established algebraic structure that has contributed greatly to the electron configurations and the molecular vibration theory. We examined several algebraic groups, that is, the finite subgroups of SL(2,C), and their corresponding chemical structures (i.e., graphs). The precise formulae of the atomic-bond connectivity index, Randić index, harmonic index, Harary index, reciprocal complementary Wiener index, the geometric–arithmetic index and its fifth version, Hosoya polynomials, and the Hosoya index were used to find the various distance- and degree-based properties of the respective graphs.

In this paper, we tried to investigate several topological properties of non-commuting graphs over finite subgroups of SL(2,C); specifically, the binary dihedral groups. However, the problem of determining the topological properties of (non-) commuting graphs, power graphs or Cayley graphs of any finite abelian or non-abelian group is still open and unresolved. An algebraic structure is essential for the development of chemical systems as well as the study of many chemical properties of molecules contained within these structures. Every index has a numerical value, and this work extends to topological indices with unique chemical structures, which may be beneficial for identifying bioactive compounds based on the physicochemical characteristics investigated in QSPR.

## Figures and Tables

**Table 1:** The following table contains a list of various topological indices.

Name of the Index	Symbol	Formula
Harary index [32]	H(Γ)	$\sum_{\left\{v,w\}\subseteq V(\Gamma \right)}\frac{1}{\left(\mathrm{dis}(v,w)\right)}$
Harmonic index [33]	$H_{r}\left(\Gamma \right)$	$\sum_{v∼w}\frac{2}{\left(d_{v}+d_{w}\right)}$
General Randić index [34]	$R_{\alpha }\left(\Gamma \right)$	$\sum_{v∼w}{\left(d_{v}\times d_{w}\right)}^{\alpha }$
Randić index [6]	$R_{-(\frac{1}{2})}\left(\Gamma \right)$	$\sum_{v∼w}\frac{1}{\sqrt{d_{v}d_{w}}}$
Reciprocal complementary Wiener index [35]	RCW(Γ)	$\sum_{\left\{v,w\}\subseteq V(\Gamma \right)}\frac{1}{\left(\mathrm{diam}(\Gamma )+1-\mathrm{dis}(v,w)\right)}$
Atomic-bond connectivity (ABC) index [35]	ABC(Γ)	$\sum_{v∼w}\sqrt{\frac{\left(d_{v}+d_{w}-2\right)}{d_{v}d_{w}}}$
Fourth version of ABC index [3]	${\mathrm{ABC}}_{4}\left(\Gamma \right)$	$\sum_{v∼w}\sqrt{\frac{\left(S_{v}+S_{w}-2\right)}{S_{v}S_{w}}}$
Geometric-arithmetic (GA) index [36]	GA(Γ)	$\sum_{v∼w}\frac{2\sqrt{d_{v}\times d_{w}}}{\left(d_{v}+d_{w}\right)}$
Fifth version of GA index [37]	$GA_{5}\left(\Gamma \right)$	$\sum_{v∼w}\frac{2\sqrt{S_{v}\times S_{w}}}{\left(S_{v}+S_{w}\right)}$
Hosoya polynomial [8]	H(Γ,x)	$\sum_{i\geq 0}\mathrm{dis}\left(\Gamma ,i\right)x^{i}$
Reciprocal status Hosoya polynomial [38]	$H_{rs}\left(\Gamma ,x\right)$	$\sum_{vw\in E(\Gamma )}x^{rs(v)+rs(w)},$ where $rs\left(w\right)=\sum_{v\in V(\Gamma ),w\neq v}\frac{1}{\mathrm{dis}(w,v)}$

Section 2 specifies every symbol used in formulas.

**Table 5:** Non-empty $K_{m}$ matchings.

$K_{m}$	$\delta_{1}$	$\delta_{2}$	$\delta_{3}$	$\delta_{4}$	⋯	$\delta_{\tau }$
$K_{2}$	22					
$K_{3}$	32					
$K_{4}$	42	124222				
$K_{5}$	52	125232				
$K_{6}$	62	126242	13624222			
$K_{7}$	72	127252	13725232			
$K_{8}$	82	128262	13826242	1482624222		
⋮	⋮	⋮	⋮	⋮	⋱	⋮
$K_{m}$	m2	12m2m−22	13m2m−22m−42	14m2m−22m−42m−62	⋯	1τ∏k=0τ−1m−2k2

## Data Availability

The data used to support the findings of this study are available within the article.
